# Acute abdominal presentation of mantle cell lymphoma mimicking acute appendicitis: a case report

**DOI:** 10.1093/jscr/rjag856

**Published:** 2026-09-30

**Authors:** Marena Gray, Ilias Marios Oikonomou, Mohammad Limbada, Stavros Gourgiotis

**Affiliations:** Department of Surgery, Addenbrooke’s CUH NHS Foundation Trust, Hills Rd., Cambridge, Cambridgeshire, CB2 0QQ, United Kingdom; Department of Surgery, Addenbrooke’s CUH NHS Foundation Trust, Hills Rd., Cambridge, Cambridgeshire, CB2 0QQ, United Kingdom; Department of Radiology, Addenbrooke’s CUH NHS Foundation Trust, Hills Rd., CB2 0QQ, Cambridge, Cambridgeshire, United Kingdom; Department of Surgery, Addenbrooke’s CUH NHS Foundation Trust, Hills Rd., Cambridge, Cambridgeshire, CB2 0QQ, United Kingdom

**Keywords:** mantle cell lypmphoma, appendicitis, emergency general surgery

## Abstract

Lymphoma presenting as an acute abdomen is rare in adults and is more commonly seen in children, particularly with Burkitt or diffuse large B-cell lymphoma. Approximately 1 per cent of appendicectomy specimens contain neoplasms, with only a very small proportion (~0.015 per cent) representing primary appendiceal lymphomas. A man in his 50s with no prior history of malignancy presented with a two-day history of right iliac fossa pain. Computed tomography demonstrated features consistent with acute appendicitis, without features suggestive of malignancy. A laparoscopic appendicectomy was performed without complication. Histopathological examination confirmed acute transmural appendicitis and mantle cell lymphoma (MCL) involving the appendiceal lymphoid tissue. The lymphoma was non-mass-forming and would likely have remained undetected without routine immunohistochemical analysis. This report describes a rare presentation of previously undiagnosed MCL identified alongside acute appendicitis, highlighting the importance of routine histopathological examination, even when intraoperative and radiological findings are consistent with uncomplicated appendicitis.

## Background

Mantle cell lymphoma (MCL) is a rare and aggressive B-cell non-Hodgkin lymphoma (NHL), accounting for ~3%–10% of adult-onset NHL in Western populations [1–3]. Lymphoma presenting as an acute abdomen is uncommon in adults and is more frequently observed in paediatric patients with Burkitt lymphoma or diffuse large B-cell lymphoma [3]. Approximately 1 per cent of appendicectomy specimens demonstrate neoplastic pathology, with only a small fraction (0.015 per cent) representing primary appendiceal lymphomas [2].

MCL typically presents with B symptoms and advanced-stage disease, often with limited diagnostic findings on initial radiological imaging [2, 4]. Consequently, only a small number of cases describing MCL presenting as an acute abdomen have been reported, each proposing differing mechanisms of pathogenesis and management strategies [4, 5]. We report an unusual case of MCL presenting as acute appendicitis prior to any oncological diagnosis or treatment.

## Case history

A man in his 50s was referred by his general practitioner to the emergency department with a two-day history of generalized abdominal pain migrating to the right iliac fossa. This was associated with fever, vomiting, and diarrhoea. His medical history included hypertension and uncomplicated diverticulosis. There was no known history of malignancy.

On examination, the patient was febrile and demonstrated localized tenderness in the right iliac fossa with no signs of generalized peritonism.

Laboratory investigations revealed a raised C-reactive protein level of 29 mg/L (reference range 0-6 mg/L) with a normal white cell count of 8.5 × 10^9^/L (reference range 3.6–10.5 × 10^9^/L). Serum amylase (25 U/L), haemoglobin (155 g/L), and lactate (1.2 mmol/L) were within normal limits.

Contrast-enhanced computed tomography (CT) imaging of the abdomen and pelvis demonstrated a retrocaecal appendix that was fluid-filled and distended to a diameter of 13 mm, with mild surrounding inflammatory changes (Fig. 1A–D). A small focus of calcification at the appendiceal tip was presumed to represent an appendicolith. There were no features of perforation or abscess formation, and only minimal free fluid was noted in the pelvis. No lymphadenopathy was reported.

**Figure 1:**
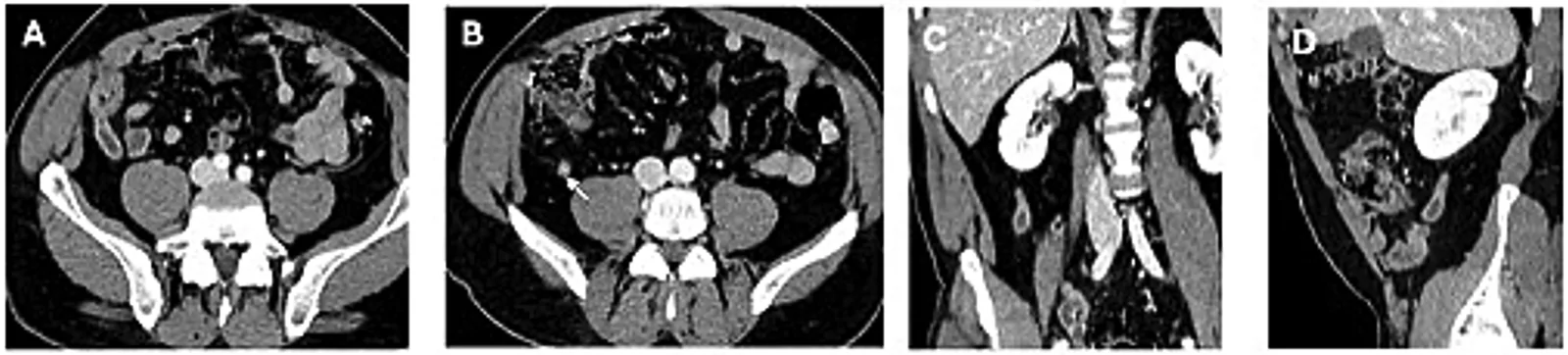
Axial (A, B), coronal (C) and sagittal (D) reformats of a contrast-enhanced CT at portal venous phase shows uncomplicated acute appendicitis, with a dilated appendix measuring up to 13 mm. There is appendiceal wall thickening, with wall hyperenhancement. There is a tiny focus of calcification at the tip, likely representing an appendicolith (white arrow). There is mild peri-appendiceal fat stranding.

The patient underwent laparoscopic appendicectomy without complication. He was discharged home two days postoperatively.

The appendix was routinely sent for histopathological analysis. Macroscopic examination revealed an appendix measuring 50 × 14 mm with a mesoappendix depth of 23 mm. The distal appendix was dilated, though the serosa was intact and smooth. On sectioning, purulent material was identified within the lumen.

Microscopic examination demonstrated acute transmural inflammation with inflammatory exudate within the lumen (Fig. 2A, thin arrow). Focally, immunoblast-like cells were identified outside the germinal centres, extending into the muscularis propria near the appendiceal tip. The lymphoid tissue showed B-cell follicles and reactive germinal centres which were surrounded by expanded mantle zones (Fig. 2A, thick arrow) containing CD20 (Fig. 2B) and Cyclin D1 (Fig. 2C and D) positive B cells. Interphase fluorescence *in situ* hybridization using Vysis probes demonstrated an IGH::CCND1 fusion in multiple nuclei within the mantle zone, confirming a diagnosis of MCL.

**Figure 2:**
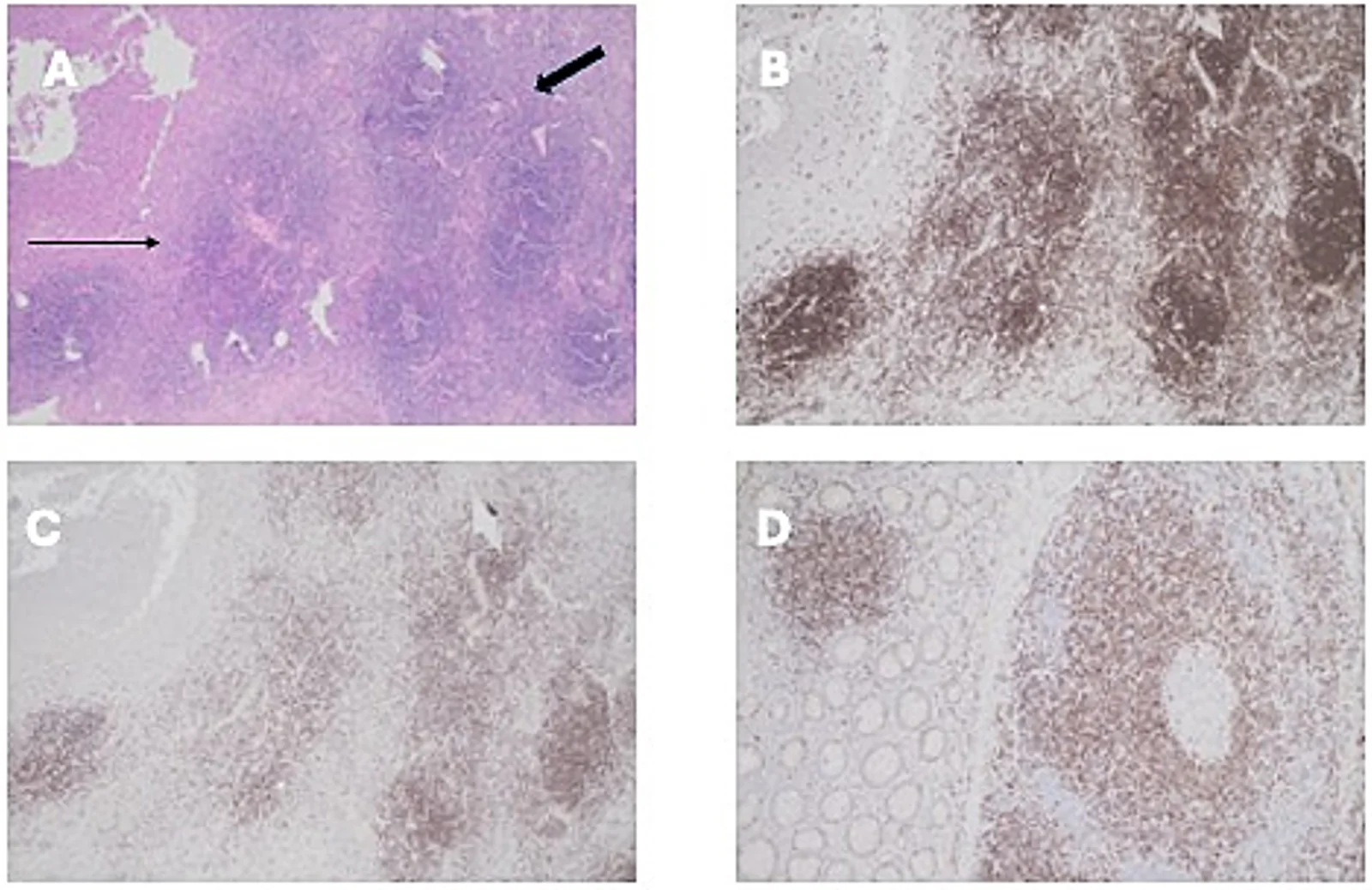
H&E, 4× showing the acute inflammation (thin arrow) and the lymphoid infiltrate (thick arrow) (A), CD20, 4× (B), Cyclin D1, 4× (C) and Cyclin D1, 10×, with Cyclin D1 positive B cells in the mantle zone surrounding a normal reactive germinal centre (D).

The patient was referred to the lymphoma multidisciplinary team and underwent further staging investigations including CT imaging, bone marrow aspiration and colonoscopy. He was diagnosed with Ann Arbor stage I MCL and received rituximab-based therapy. Post-treatment bone marrow assessment demonstrated no evidence of clonal B-cell infiltration. The patient completed treatment in August 2025 and remains under haematology follow-up. At approximately eight months following completion of therapy, there was no clinical evidence of disease recurrence or progression.

## Discussion

MCL is an aggressive intermediate-grade B-cell neoplasm composed of monomorphic small- to medium-sized lymphocytes, characterized by overexpression of Cyclin D1 secondary to the t(11;14)(q13;q32) translocation [1, 6]. These neoplastic cells originate from the mantle zone of lymphoid follicles.

Patients typically present with advanced disease, including generalized lymphadenopathy and frequent extranodal involvement of the bone marrow, spleen, and gastrointestinal tract [2, 5]. Compared with other B-cell lymphomas, MCL is associated with reduced long-term survival and early relapse despite systemic therapy [2].

The diagnosis of MCL relies on histopathological examination and immunohistochemical analysis, with differentiation between *in situ* mantle cell neoplasia and overt MCL being essential [1, 6, 9]. In patients with gastrointestinal involvement, endoscopy and colonoscopy are valuable diagnostic tools, particularly with advances in endoscopic ultrasonography facilitating detection and biopsy of submucosal lesions [5].

Surgical intervention in MCL is uncommon and generally reserved for complications such as bowel obstruction, perforation, bleeding, or appendicitis [4, 5, 9]. Appendiceal involvement by lymphoma is rare and most commonly associated with diffuse large B-cell lymphoma or Burkitt lymphoma [3, 4, 9]. Proposed mechanisms include luminal obstruction by lymphomatous infiltration or extrinsic compression due to lymphomatous polyposis [4, 5].

Radiologically, appendicitis is typically diagnosed based on a combination of features, such as when the appendiceal diameter exceeds 6 mm, wall thickness exceeds 3 mm, alongside associated inflammatory changes [4, 9]. An appendiceal diameter greater than 30 mm should raise suspicion for appendiceal lymphoma, particularly in the presence of regional lymphadenopathy, however these were absent in this case [4, 9]. Current literature does not demonstrate a survival benefit of right hemicolectomy over appendicectomy alone in these cases [4]. Accordingly, a routine appendicectomy was appropriate given the absence of caecal involvement.

In the present case, complete luminal occlusion was not observed; however, transmural inflammation and intraluminal exudate were evident. While appendicitis has been reported in patients with known MCL following chemotherapy—often in the context of neutropenia—this was not a contributing factor in our patient [5]. The MCL identified within the appendiceal lymphoid tissue was not mass-forming and was relatively subtle on routine microscopy, becoming apparent only following immunohistochemical staining. In this context, the lymphoma was likely an incidental finding coexisting with acute appendicitis rather than the primary cause of luminal obstruction.

The role of routine histopathological examination following appendicectomy has been debated, with some advocating selective submission based on macroscopic findings [7, 8]. In this case, there were no radiological or intraoperative features to suggest malignancy. A selective policy based on macroscopic appearance alone would likely have resulted in missed diagnosis of early-stage MCL. This case therefore supports continued routine pathological evaluation of appendicectomy specimens.

## Conclusions

This case demonstrates histologically confirmed acute appendicitis with incidental MCL. Although appendiceal involvement by lymphoma is uncommon, this reinforces the importance of routine histopathological examination of all appendicectomy specimens, as clinically unsuspected malignant pathology may be present despite typical presentation. Early recognition of atypical pathological features allows timely referral for staging and oncological management, which may significantly influence patient outcomes. MCL should be considered in the differential diagnosis of acute abdominal presentations, particularly in middle-aged male patients.
